# Cardiac Autonomic Nervous System and Ventricular Arrhythmias: The Role of Radionuclide Molecular Imaging

**DOI:** 10.3390/diagnostics11071273

**Published:** 2021-07-15

**Authors:** Andreas Fesas, Evanthia Giannoula, Alexis Vrachimis, Argyrios Doumas, Christian Wenning, Matthaios Didagelos, Ioannis Iakovou

**Affiliations:** 1 Department of Nuclear Medicine, German Oncology Center, University Hospital of the European University, 4108 Limassol, Cyprus; Andreas.Fesas@goc.com.cy (A.F.); alexis.vrachimis@goc.com.cy (A.V.); 2 Second Academic Nuclear Medicine Department of Aristotle University, AHEPA Acad. Hospital, 54636 Thessaloniki, Greece; eva_giann@hotmail.com (E.G.); drdumas@the.forthnet.gr (A.D.); 3 C.A.R.I.C. Cancer Research & Innovation Center, 4108 Limassol, Cyprus; 4 Department of Nuclear Medicine, Bonifatius Hospital gGmbH, 49808 Lingen, Germany; Christian.Wenning@hospital-lingen.de; 5 1st Cardiology Department, of Aristotle University, AHEPA Acad. Hospital, 54636 Thessaloniki, Greece; manthosdid@yahoo.gr

**Keywords:** PET 1, SPECT 2, arrhythmias 3, autonomous nervous system imaging 4, radiopharmaceuticals 5

## Abstract

Widely established compared to myocardial perfusion imaging, cardiac autonomous nervous system (CANS) assessment by radiopharmaceutical means is of potential use especially to arrhythmogenic diseases not correlated with anatomic or functional alterations revealed by classical imaging techniques. Molecular imaging of both pre- and postsynaptic functions of the autonomous nervous system is currently feasible, since single photon emission tomography (SPECT) and positron emission tomography (PET) have the ability to reveal the insights of molecular pathophysiology depicting both sympathetic and parasympathetic imbalance in discrete heart pathologies. This review provides not only a brief presentation of radiopharmaceuticals used for non-invasive CANS imaging in the case of ventricular arrhythmias, but also a current update on ventricular tachycardias, cardiomyopathies, Brugada and Long QT syndrome literature.

## 1. Introduction

The autonomous nervous system provides cardiac dynamic control during the entire life. A multilevel neural network is responsible for control of chronotropy, lusitropy, dromotropy, and inotropy function. Ventricular arrhythmogenesis is commonly attributed to impaired autonomic innervation and pre/postsynaptic imbalance [1]. Nuclear cardiac imaging is the only method for the non-invasive evaluation of the functional integrity of cardiac innervation. Radiopharmaceutical techniques have been used for decades now, for functional imaging of the cardiac sympathetic and parasympathetic tone. During the last decade, several improvements and developments have been made on the radiopharmaceutical approach and synthesis routes yet, most attention has been given to the sympathetic arm of the nervous system [2,3]. For example, in order to target the neurotransmitter norepinephrine, the single photon emission tomography (SPECT) radiotracer [^123^I]- metaiodobenzylguanidine ([^123^I]-MIBG) and the positron emission tomography (PET) radiotracer [^11^C]-meta-hydroxyephedrine ([^11^C]-mHED) were developed. Both radiopharmaceuticals depict cardiac sympathetic activity by mimicking the neuronal transport and storage process of norepinephrine. A radiotracer’s success is dependent upon its behavioral similarities to the endogenous neurotransmitter.

The parasympathetic arm of the heart nervous system primarily uses acetylcholine (ACh) and alternatively peptides as the physiological neurotransmitters. *N*-[^11^C]- methylquinuclidinyl benzilate ([^11^C]-MQNB) has been the tracer with the highest affinity towards muscarinic acetylcholine receptors and is considered the best representative of radiopharmaceuticals assessing the parasympathetic arm of the cardiac autonomous nervous system [4].

These radiotracers can be categorized into three main categories, the labelled neurotransmitters such as [^18^F] dopamine and [^11^C] epinephrine, the substrate analogues such as [^123^I]-MIBG, [^11^C]-mHED and [^11^C]-phenylephrine and uptake-1 inhibitors such as [^11^C]-methylreboxetine [4]. Each one of the aforementioned radiotracers exhibits unique characteristics, giving different information on the function of the cardiac nervous system. Neuronal uptake, vesicular concentration and release as well as catecholamine metabolism, are only some of the information that can be obtained. Several tracers have been developed with various chemical and physical properties and characteristics, in an attempt to develop the ideal tracer. Despite the attention the sympathetic arm of the nervous system receives, radiotracers targeting the parasympathetic arm of cardiac nervous system have been used mainly on a research base. Herein, the most commonly used radiotracers in clinical practice are assessed together with the promising radiotracers for imaging the cardiac autonomous nervous system in several kind of cardiomyopathies, like left and right ventricular tachycardias, Long QT syndrome and Brugada syndrome.

## 2. Single Photon Emission Tomography (SPECT) Radiotracers

**Figure 1:**
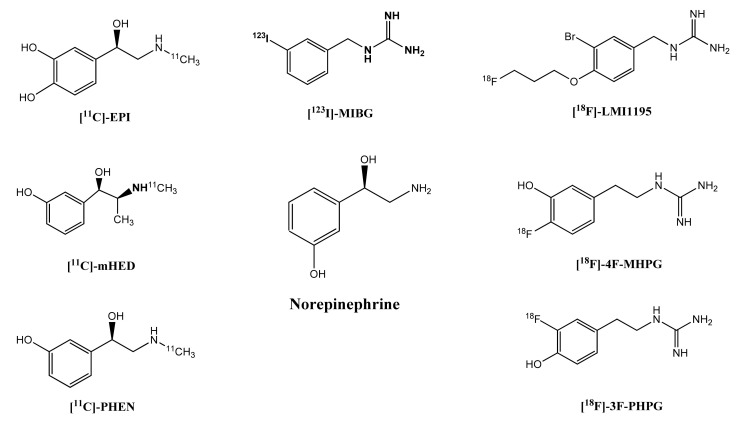
Chemical structures of the norepinephrine and its radiolabeled analogues. ([^11^C]-EPI:[^11^C]-Epinephrine, [^123^I]-MIBG: [^123^I]-metaiodobenzylguanidine, [^18^F]-LMI1195: *N*-[3-bromo-4-(3-[^18^F]fluoro-propoxy)-benzyl]-guanidine, [^11^C]-mHED: [^11^C]-meta-hydroxyephedrine, [^11^C]-PHEN: [^11^C]-phenylephrine, [^18^F]-4F-MHPG: 4-[^18^F]-fluoro-3-hydroxy-phenelthylguadinine, [^18^F]-3F-PHPG: 3-[^18^F]-fluoro-4-hydroxy-phenelthylguadinine).

[^123^I]-MIBG (Figure 1) is considered the gold standard and the radiopharmaceutical of choice for the examining the presynaptic function of sympathetic innervation, being the most widely used radiotracer for that purpose during the last 30 years [5,6]. Originally was developed as a diagnostic agent for adrenal medulla however, due to its norepinephrine similarities, increased tracer uptake was demonstrated in the heart and salivary gland as well as in tumors expressing neurohormone receptors. The first use in humans was in 1981 by University of Michigan investigators [7]. [^123^I]-MIBG myocardial scintigraphy has been proven to be a powerful tool for patients suffering from cardiomyopathies and heart failure [8,9].Several radiolabeling methods were used in the past, trying to improve the radiochemical yield, as well as the specific activity of radioiodinated MIBG [10,11,12].

## 3. Positron Emission Tomography (PET) Radiotracers

### 3.1. ^11^C-Labeled Radiotracers

Currently, several norepinephrine ^11^C-labelled analogues are available and under investigation. [^11^C]meta-Hydroxyephedrine ([^11^C]mHED, Figure 1) is one of them and is the most widely used PET radiotracer for imaging the presynaptic function of the sympathetic nervous system [4,6,13]. [^11^C]mHED is rapidly transported into sympathetic neurons by the norepinephrine transporters by uptake-1 and stored in vesicles. It is not susceptible to monoamine oxidase (MAO) breakdown [14,15,16]. Hydroxyephedrine is synthesized by N-methylation of metaraminol which is a synthetic false neurotransmitter of norepinephrine. 

#### 3.1.1. [^11^C]-Epinephrine ([^11^C]-EPI)

[^11^C]-EPI (Figure 1) is structurally identical to the endogenous neurotransmitter and is the second most common radiopharmaceutical for imaging of the sympathetic nervous system using PET [4,17]. It can be synthesised from norepinephrine by direct N-methylation reporting high radiochemical purity and yield and high specific activity. Due to its more natural behaviour than [^11^C]mHED and its susceptibility to MAO metabolism, lower overall washout rates and decreased loss of tracer due to passive diffusion have been reported [16].

#### 3.1.2. [^11^C]-Phenylephrine ([^11^C]-PHEN)

[^11^C]-PHEN (Figure 1) is a synthetic catecholamine analogue, and in contrast to mHED is susceptible to MAO metabolism. Even though it exhibits selectivity for uptake-1, show greater permeability than both mHED and EPI due to its lack of α-methyl group [18,19].

#### 3.1.3. [^11^C]-*N*-Methylquinuclidin-3-ylbenzilate([^11^C]-MQNB)

[^11^C]-MQNB is considered the best representative of radiopharmaceuticals assessing the parasympathetic arm of the cardiac autonomous nervous system. It a hydrophilic, non-metabolized and highly specific cholinergic muscarinic antagonist [20]. [^11^C]-MQNB is high yield and specific activity synthesized, with great reliability. Synthesis of the radiotracer is achieved by N-methylation of the QNB precursor.

### 3.2. ^18^F-Labeled Radiotracers

Despite the advantages that carbon-11 radiopharmaceuticals such as HED have, their short half-life, of 20 min, limit the flexibility on their transportation, leading to the necessity of on-site cyclotron productions. As a result, both the designing of new radiotracers and the price of the existing radiopharmaceuticals are affected. On the other hand, fluorine-18 has a longer half-life of 110 min which is considered ideal for the design and development of new radiotracers, and can be dispatched from a central production site to several smaller facilities for administration. The aforementioned model of a central production site has been established for the production and use of ^18^F-fluorodeoxyglucose (FDG) and it is proven to be cost effective. As a result, several attempts for designing ^18^F labelled traces for imaging the sympathetic nervous system have been made.

#### 3.2.1. *N*-[3-Bromo-4-(3-[^18^F]fluoro-propoxy)-benzyl]-guanidine ([^18^F]-LMI1195)

[^18^F]-LMI1195 (Figure 1) is so far the most promising tracer for commercialization since it has entered a phase I clinical trial [6] quite recently exhibiting favorable biodistribution for cardiac imaging with acceptable radiation doses. It is a norepinephrine analogue and it is taken up via uptake-1 mechanism [21]. It has a benzylquanidine-based structure, similarly to [^123^I]-MIBG, and it is not metabolised by cytosolic monoamine oxidase (MAO).

#### 3.2.2. 6-[^18^F]-Dopamine (6F-DA)

6F-DA can be used for imaging the function of cardiac sympathetic innervation. It exhibits almost identical properties to an endogenous dopamine neurotransmitter with respect to uptake and release kinetics and metabolism [3]. Dopamine converts to norepinephrine within the presynaptic neuron and is susceptible to further metabolism. 

#### 3.2.3. [^18^F]-4F-MHPG (4-[^18^F]-fluoro-3-hydroxy-phenelthylguadinine) 

[^18^F]-4F-MHPG (Figure 1) was designed in an attempt to achieve slow cardiac uptake, combined with extended retention, for distinguishing mild changes of sympathetic nervous system, excluding at the same time the blood flow effect [22]. In order to optimize the properties of the tracer, its isomer 3-[^18^F]-fluoro-4-hydroxy-phenelthylguadinine ([^18^F]-3F-PHPG) was produced and investigated. 

## 4. Cardiomyopathies

According to the most recent definition provided by the European Society of Cardiology in 2008: “Cardiomyopathy is a myocardial disorder in which the heart muscle is structurally and functionally abnormal, in the absence of coronary artery disease, hypertension, valvular disease and congenital heart disease sufficient to cause the observed myocardial abnormality” [23]. Cardiomyopathies are classified into distinct morphological and functional phenotrypes (hypertrophic, dilated, restrictive, arrhythmogenic, unclassified). Each phenotype can be further subdivided into familial/genetic and non-familial/non-genetic forms [20,23]. The most common cardiomyopathies often present with similar symptoms, such as fatigue, peripheral edema, dyspnea or orthopnea, paroxysmal nocturnal dyspnea, presyncope, syncope, and finally cardiac ischemia [24]. Hypertrophic cardiomyopathy (HCM) represents the most commonly seen form of inherited cardiomyopathy, whilst other types, like arrhythmogenic right ventricular cardiomyopathy (ARVC) are somehow rare. It has been demonstrated that cardiac [^123^I]-MIBG uptake often decreases in patients with cardiomyopathies, revealing alterations in the adrenergic innervation in the heart of these patients [25,26,27]. These observations provided the incentive for further investigation of the link between cardiomyopathies and myocardial autonomic disorders, as well as their pathophysiology background, especially left ventricular (LV) function and perfusion.

### 4.1. Dilated Cardiomyopathy (DCM)

Dilated cardiomyopathy (DCM) remains a major cause of sudden cardiac death (SCD) and heart failure (HF). It is characterized by left ventricular (LV) dilatation and systolic dysfunction (while right ventricular dilatation and dysfunction can also coexist but are not mandatory for the diagnosis) in the absence of other conditions (as defined in the cardiomyopathies definition) that could lead to global systolic impairment [23]. Familial forms usually follow an autosomal dominant pattern of inheritance and non-familial forms could be due to cardiac inflammation, nutritional or endocrine disorders and exposure to cardiotoxic drugs [23,28]. Given the poor prognosis of DCM, it is imperative to develop accurate, non-invasive diagnostic methods to evaluate the severity of alterations in cardiac autonomic innervation [29]. Cardiac autonomic imaging by means of SPECT and PET may help to accurately and non-invasively identify patients with DCM.

Although adrenergic derangement in patients with cardiomyopathies has been described since the 1980s [30], the relationship between sympathetic denervation and the occurrence of ventricular tachycardia (VT) in patients with DCM was initially described by Maeno et al. in 1993. The study group showed focal sympathetic innervation defects (with [^123^I]-MIBG) at the left ventricular myocardial walls, while the perfusion was preserved in patients with idiopathic DCM. Despite the small number of patients who had been examined, Maeno et al. laid down the foundations for the identification of patients with DCM at risk for the development of VT [31]. Except for arrhythmias in DCM, several studies have been conducted showing significant association between [^123^I]-MIBG uptake and washout index and LV function, perfusion and histopathologic abnormalities in patients with DCM. Yakamado et al. investigated changes in myocardial [^123^I]-MIBG concentrations in patients with DCM, by comparing myocardial uptake ratio (MUR) both for MIBG and ^201^Tl. They found that the washout of [^123^I]-MIBG, was significantly increased in DCM compared to the control group, reflecting the severity of the disease in these patients. The association between cardiac sympathetic innervation and function, LV function and perfusion in cardiomyopathy was investigated by Zhao et al., concluding that quantifying MIBG washout may prove myocardial functional impairment [27,32,33]. Moreover, the prognosis of DCM has been approached by means of MIBG. Yamazaki et al. evaluated the severity and prognosis of DCM using [^123^I]-MIBG). They showed that patients suffering from DCM, and appearing with high [^123^I]-MIBG uptake during early imaging, are highly possible to improve by beta-blocker medication, suggesting that this method could be appropriate for evaluating DCM severity, usefulness and right dosage of beta-blocker therapy, and prognosis [34].

Although, there are several studies that examine the myocardial autonomous intervention by PET imaging in DCM, to the best of our knowledge there are no clinical trials evaluating the risk for arrhythmias in DCM using PET autonomous myocardial imaging. Guludec et al. found increased myocardial muscarinic receptor density in patients with idiopathic DCM using PET with [^11^C]-MQNB). Moreover, PET has been proved to play a role in evaluating the association of occult inflammation in DCM and ventricular arrhythmias. Approximately 50% of patients referring to the UCLA Cardiac Arrhythmia Center from 1/2012 to 1/2015, suffering from cardiomyopathy of unknown origin and ventricular arrhythmias (VA) demonstrated on FDG PET persistent focal myocardial inflammation [35]. These data suggest that a significant proportion of patients labeled as “idiopathic” may have an occult arrhythmogenic inflammatory substrate, making them suitable candidates for immunosuppression therapy. Thus, nuclear imaging in DCM can serve both as a diagnostic and a prognostic tool [35].

### 4.2. Hypertrophic Cardiomyopathy (HCM)

Hypertrophic cardiomyopathy (HCM) is characterized by increased LV wall thickness unexplained by abnormal loading conditions (hypertension, aortic valve stenosis etc.) and systemic diseases such as amyloidosis and glycogen storage disease. It is the most common genetic cardiovascular disorder, following an autosomal dominant inheritance, with high clinical heterogeneity [29]. The prevalence of HCM in the general population worldwide is estimated at 0.2% [36]. Left ventricular outflow tract obstruction may or may not be present. Even though HCM patients remain mainly asymptomatic, the disease is characterized by an increased risk of VA and unfortunately sudden death due to malignant arrhythmias can be usually the first clinical presentation, especially in young adults and athletes [37].

Several other studies concerning HCM, dealing with myocardial sympathetic imaging, showed that MIBG is useful for the evaluation of sympathetic innervation and activity in HCM [38]. More than 30 years ago Nakajima et al. showed that in patients with severe septal hypertrophy (greater than 20 mm), the MIBG clearance was significantly higher compared with less hypertrophic (less than or equal to 20 mm) group [39]. Pace et al. performed [^123^I]-MIBG scintigraphy, [^99m^Tc]-MIBI SPECT at rest, and echocardiography in 11 patients with HCM in order to assess the relationship between sympathetic nervous function and left ventricular perfusion and function. Similarly, they found positive relationship of [^123^I]-MIBG washout rate with septum thickness. Moreover, they showed a positive relationship between MIBG clearance and left ventricular outflow tract gradient and negative one with left atrial fractional shortening. Their results showed close correlation of the myocardial sympathetic activity to cardiac anatomy (such as degree of hypertrophy) as well as diastolic function in patients suffering from HCM [40]. Isobe et al. stated that in patients with HCM there is limited information about the relationship between left ventricular functional reserve seen during exercise and cardiac sympathetic innervation. In this context they examined 30 HCM patients using [^123^I]-MIBG scintigraphy and ECG at rest and after biventricular cardiac catheterization at rest and during exercise. They concluded that in patients with non-obstructive HCM, LV functional reserve in response to exercise can be non-invasively evaluated at rest using [^123^I]-MIBG myocardial scintigraphy [41]. The knowledge towards the relationship between MIBG concentration and clearance with impairment of cardiac sympathetic innervation and LV function in HCM, although based on small clinical studies is well established. However, the potential role of MIBG imaging for identifying sustained ventricular tachycardia in HCM is much less investigated.

Terrai et al. evaluated the relationship between cardiac sympathetic innervation and the rate of VT in HCM patients. They performed MIBG scintigraphy and 24-h ambulatory electrocardiographic monitoring in 44 HCM patients, 15 with ventricular tachyarrhythmia (group A) and 29 without ventricular tachyarrhythmia (group B). They showed that the washout rate was significantly higher in group A than in group B, concluding that occurrence of malignant ventricular tachyarrhythmia in HCM is associated with global cardiac sympathetic nerve activity rather than with the heterogeneity of this activity [42]. 

Although, cardiac sympathetic innervation using PET tracers is increasingly investigated, its contribution on predicting the risk for arrhythmias has been studied to a much lesser extent [43]. Li et al. studied left myocardial perfusion and sympathetic nerve function in normal subjects and in patients with HCM, using [^13^N]-ammonia (^13^NH_3_) PET scanning.6-[^18^F]-fluorodopamine (^18^F-FDA) served as the sympathetic neuronal imaging agent in 8 patients with HCM and 15 healthy controls. Their results stated that myocardial [^13^NH_3_]-imaging was similar in hypertrophied and non-hypertrophied myocardium in both HCM patients and in normal volunteers. The ^18^F/^13^N ratio was inferior in hypertrophied than in non-hypertrophied HCM regions, as well as in the septum of normal volunteers. These results proved that in HCM there is decreased neuronal uptake of catecholamines in hypertrophied but not in non-hypertrophied myocardium. By enhancing norepinephrine delivery to adrenoceptors for a given amount of sympathetic nerve traffic, decreased neuronal uptake could explain major clinical features of HCM such as arrhythmias [44].

### 4.3. Arrhythmogenic Right Ventricular Cardiomyopathy

Arrhythmogenic right ventricular cardiomyopathy (ARVC), is a rare inherited disorder, which is characterized by cardiac electrical instability due to fibro fatty degeneration of the right and/or left ventricular myocardium. It presents clinically with right ventricular dysfunction (global or regional), with or without left ventricular disease. Sudden death, especially in young athletes due to tachyarrhythmias or right-heart failure may be seen [45]. Provocation of ventricular tachycardia may evolve frequently and especially during exercise due to elevated catecholamines sensitivity, a fact that suggests the involvement of the sympathetic innervation in the development and maintenance of arrhythmias in patients with (ARVC). The problem is that arrhythmias originate usually from the right ventricle, which cannot be imaged adequately by SPECT or PET, as is the case with the left ventricle [5].

Originally Lerch et al. showed that using [^123^I]-MIBG scintigraphy in ARVC patients and no morphological or functional left ventricular abnormalities allows detection of adrenergic dysinnervation of the left ventricle. Twenty-two of the 25 patients that they enrolled showed reduced uptake of MIBG, while [^201^Tl] SPECT was normal in 16 patients. The remaining 9 patients showed areas of slight hypoperfusion not correlated with the reduced MIBG uptake [46]. A year later Wichter et al. extracted similar results; 40 ARVC patients (83%) demonstrated regional reductions or defects of MIBG uptake, while all healthy individuals demonstrated a uniform radio pharmaceutical uptake at the left ventricular wall. The vast majority of the ARVC patients (95%) and abnormal MIBG scintigram, showed reduced tracer uptake at the basal and posteroseptal wall of the left ventricle. Some involvement of the adjacent lateral wall was evident in 10 patients, while the anterior and the apex in 2 and 12 respectively. The right ventricle was not visible in any patient on MIBG scan and therefore its innervation could not be evaluated. As a result, the authors postulated that in the early diagnosis of ARVC cardiac sympathetic innervation may play a major role to these patients [47]. Other papers indicate that a reduction of MIBG uptake at the left ventricular walls is associated with a higher risk of future recurrence of life-threatening ventricular tachyarrhythmias, independently of the extent of right ventricular dysfunction [41]. In their recent study Todica et al. highlighted the limitations of previous works which did not consider right ventricle’s uptake, suggesting that the diagnosis of ARVC should be better supported by quantitation of right ventricular [^123^I]-MIBG uptake. To the best of our knowledge this study group is the only one to employ state-of-the-art functional SPECT/CT hybrid imaging to evaluate right and left ventricle uptake separately. They found a significant reduction [^123^I] MIBG uptake at the area of the right ventricular wall in ARVC patients compared to their reference group (with idiopathic ventricular fibrillation), such that the right ventricle/mediastinum ratio (RV/M) confirmed diagnosis of ARVC with a high sensitivity and specificity [48].

As a matter of fact, most studies aim to demonstrate abnormalities of the presynaptic uptake-1 assessed by MIBG. However, Wichter et al., investigated neuronal reuptake of norepinephrine and beta-adrenergic receptor density by means of PET/CT in patients with ARVC. In eight patients diagnosed with ARVC they performed PET with [^11^C]-HED to assess presynaptic norepinephrine reuptake, with [^11^C]CGP-12177 to assess postsynaptic beta-adrenergic receptor density, and with [^15^O]H_2_O for quantification of myocardial blood flow. They found a significant reduction of myocardial beta-adrenergic receptor density in patients with ARVC in comparison with age-matched control subjects. That was attributed to secondary downregulation of β-adrenergic receptors, due to locally increased synaptic norepinephrine levels. This may be the result of either an increased sympathetic tone or because of impaired presynaptic catecholamine reuptake [49]. Even though we have some promising initial results, in the field of imaging cardiac sympathetic innervation, the potential implication for the arrhythmia profile between other competing modalities for risk stratifying patients with ARVC still remains to be elucidated by larger prospective trials [5].

### 4.4. Ischemic Cardiomyopathy

Ischemic cardiomyopathy (ICM) describes dysfunction of left ventricular contractility as a result of ischemic damage to the myocardium (most commonly due to coronary artery disease), that leads to cell death, fibrosis, left ventricular enlargement and dilation. The autonomic nervous system is a key modulator of ischemic arrhythmogenesis [50]. Previous studies have shown a correlation between sympathetic activation and lethal ventricular arrhythmias. Inhomogeneity in myocardial sympathetic innervations as a result of infarction or reversible ischemia may create a myocardial substrate particularly vulnerable to arrhythmic death [51]. Moreover, sympathetic denervation affects not only the area of infarction, but also exceeds that of infarction following coronary artery occlusion. These regions adjacent to an area of infarction as well as hibernating myocardium may be particularly arrhythmogenic. This is the result of downstream denervation from irreversible injury to the sympathetic nerves traversing the area of infarction as they course from the base of the heart toward the apex [52]. 

One way to assess myocardial autonomic function in ICM is with MIBG imaging. Quantitatevely, cardiac [^123^I]-MIBG uptake can be expressed, by the heart-to-mediastinum ratio (H/M), that has independent and incremental prognostic value, being able to identify high risk ICM patients. However, the long-term prognostic value of MIBG uptake in patient with ischemic versus idiopathic cardiomyopathy remains unclear [53]. Wakabayashi et al.in a prospective study using quantitative MIBG scintigraphy, in 76 ischemic and 56 idiopathic cardiomyopathy patients who were followed up for 54 months showed that late H/M was the best independent predictor of cardiac death. However, this was valid for both ischemic and idiopathic cardiomyopathy with no distinct differences, suggesting that both diseases share common pathophysiologic and prognostic manifestations of a malfunctioning cardiac sympathetic innervation [54]. Further results have indicated potential trigger-substrate interaction and the vulnerability for ventricular arrhythmias based on MIBG H/M. Avendaño et al. examined acute psychological stressors as precursors for ventricular arrhythmias in patients with ICM. Both ICM patients and control group underwent [^123^I]-MIBG and [^99m^Tc]-Tetrofosmin SPECT/CT imaging during an anger recall mental stress task and dual isotope imaging was repeated approximately 1 week later during rest. They found that the hemodynamic response to mental stress was similar in both groups. Anger recall significantly decreased the MIBG H/M in ICM patients (2.62 ± 0.3, *p* = 0.04), but not in normal subjects, which is indicative of the pathophysiological mechanisms causing arrhythmia in ICM under certain trigger [55]. Boogers et al. investigated the assumption that cardiac MIBG scintigraphy may be the predictor of future ventricular arrhythmias leading to ICD therapy. He studied 116 heart failure patients referred for ICD therapy. MIBG scintigraphy was performed before ICD implantation. During a mean follow-up of 23 ± 15 months, the primary end point (defined as appropriate ICD therapy = ventricular arrhythmia correctly treated by the ICD) occurred in 21% of the patients while the secondary end point (combined outcome of appropriate ICD therapy or cardiac death) in 28% of the patients. Late MIBG SPECT defect score was the only independent predictor for both end points. Patients appearing with larger MIBG defects had significantly more appropriate ICD therapy and/or cardiac death, compared to individuals with smaller innervation defects [56].

The PAREPET (Prediction of Arrhythmic Events with Positron Emission Tomography) study was designed to determine whether quantifying inhomogeneity in myocardial sympathetic innervation by PET imaging could identify ICM patients at highest risk for sudden cardiac arrest (SCA). It prospectively enrolled 204 ICM patients who underwent PET imaging to quantify cardiac sympathetic denervation ([^11^C]-mHED), perfusion ([^13^N]-ammonia) and viability (18-FDG). Myocardial sympathetic denervation quantified by [^11^C]-mHED PET was able to identify ICM patients at high risk of SCA. The total volume of denervated myocardium was the main risk factor of SCA and was independent of other imaging parameters like hibernating myocardium or Left Ventricular Injection Fraction (LVEF). These results indicate that being able to quantify the extent of cardiac sympathetic denervation may help significantly in the identification of ICM patients who will benefit the most from a primary prevention ICD implantation [57].

## 5. Arrhythmogenic Non-Cardiomyopathy Diseases

### 5.1. Idiopathic Right Ventricular Outflow Tract Tachycardia

Observed VT can be associated with structural heart disease. However, there are cases of patients with VT, experiencing no such issues or other abnormalities or long QT syndrome. These cases are estimated to be at about 10% of all observed VTs. Outflow tract VT (OTVT) include a subset of idiopathic VT, which are predominantly located both in and around the right and left ventricular outflow tracts. The most common type of idiopathic VT remains the right ventricular outflow tract VT (RVOT VT). It manifests at a relatively early age (30–50 years) and it is more common in females. Phenotypically, RVOT tachycardia can be distinguished or divided into two predominant subsets, one that shows repetitive monomorphic non-sustained VT and the other which displays paroxysmal exercise induced sustained VT [58]. Frequently, inducible tachyarrhythmias under physical or mental stress and/or during cateholamine infusion, are suggestive of primary involvement of cardiac innervation abnormalities (cardiac neuropathy). Moreover, these arrhythmias can be successfully suppressed by anti-adrenergic drugs [59]. Based on this hypothesis, Schäfers et al. assessed local presynaptic norepinephrine re-uptake the norepinephrine analog MIBG in 45 patients with idiopathic RVOT-VT, 25 patients with idiopathic LVOT-VT, 15 idiopathic ventricular fibrillation (IVF)-patients and 10 control at comparable age range. They showed locally reduced uptake in 27 of 45 RVOT-VT patients (60%), 5 of 15 LVOT-VT patients (33%) and 17 of 25 IVF patients (68%), proving presynaptic sympathetic dysfunction in OT VT [60].

Schäfers et al. investigated patients with RVO-VT for both the reuptake of norepinephrine (uptake-1) in the neurons and the beta-adrenoceptor density, by means of PET. Using [^11^C]-mHED and PET imaging, they examined the presynaptic reuptake of norepinephrinein 8 patients with idiopathic RVO-VT and in a total of 29 control patients of the same age range. Additionally, [^15^O]-H_2_O was used to quantify the myocardial blood flow and [^11^C]CGP 12177 to measure the postsynaptic beta-adrenoceptor density. It was proven that both beta-adrenoceptor density and myocardial catecholamine reuptake were significantly decreased in patients with idiopathic RVO-VT, suggesting that in patients with RVO-VT, downregulation of myocardial beta-adrenoceptor occurs in conjunction to an increase in of local synaptic catecholamines, due to impaired catecholamine reuptake [61]. To the best of our knowledge, there are no prospective studies evaluating the prognostic value using sympathetic myocardial innervation imaging, in patients with RVOT.

### 5.2. Idiopathic Left Ventricular Tachycardia and Idiopathic VentricularFibrillation

Idiopathic left ventricular tachycardia (ILVT) is a ventricular arrhythmia observed in the absence of structural heart disease caused mainly due to re-entry at the fascicles of the left ventricle. It is further divided into three sub-types: left posterior fascicular Vt, left anterior fascicular VT high septal fascicular VT. It affects young patients (15–40 years) and predominately males (>60%) [62].

Idiopathic ventricular fibrillation (IVF) is a life-threatening ventricular arrhythmia, presenting as syncope or SCD in young people with with apparently normal hearts and no identifiable genetic syndromes [62]. Involvement of the adrenergic system in the pathogenesis of IVF has been documented by several researchers [60].

Sympathetic denervation has been demonstrated scintigraphically in patients with cardiomyopathies, after myocardial infarction or long QT syndrome. For the first time, Mitrani et al. investigated the role of abnormalities in cardiac sympathetic innervation in the pathogenesis of VT in the absence of coronary artery disease. They performed cardiac MIBG and ^201^Tl SPECT scans at rest in 18 cardiomyopathy patients, with left ventricular hypertrophy, valvular disease or a structurally normal heart, who experienced monomorphic or polymorphic ventricular tachycardia. A group of 12 patients with cardiomyopathy or a structurally normal heart, without ventricular tachycardia served as controls. Among other results, they found that 55% of the patients with ILVT had myocardial areas with normal ^201^Tl uptake but decreased or minimal [^123^I]-MIBG uptake. On the other hand nobody from the control group had these characteristics [59]. The asymmetry between cardiac MIBG and thallium scans in patients with ventricular tachycardia in patients with “clinically and structurally normal” heart, was also investigated by Gill et al., supporting the hypothesis that selective denervation in the septal portion of the left ventricle, leading to an imbalance of the sympathetic supply to the myocardium and locally imbalanced sympathetic or parasympathetic interactions, is an important mechanism in the genesis of VT in the absence of structural heart disease [63].

The potential impact of sympathetic dysfunction on the long-term prognosis of patients with IVF was examined by [^123^I] MIBG SPECT by Paul et al. The study group performed MIBG scans in 20 patients (as well as in a control group of 10). The follow-up time was 7.2+/−1.5 years (range 4.9–10.5 years). During follow-up, 18 episodes of VF/fast polymorphic ventricular tachycardias appeared in 4 IVF patients with abnormal MIBG uptake, while only 2 episodes of monomorphic ventricular tachycardia occurred in a single IVF patient with normal MIBG uptake. Their study revealed that future episodes of life-threatening ventricular tachyarrhythmias were more likely to occur in patients with IVF and impaired sympathetic innervation [56]. The literature review conducted for the purposes of the current paper did not reveal any larger scale study or any study investigating adrenergic myocardial innervations impairments in patients with ILVT or IVF in the absence of structural heart disease. 

### 5.3. Brugada Syndrome

Brugada syndrome (BS) is classified to the inherited primary arrhythmia syndromes, a group of heterogeneous conditions, that appear in structurally normal hearts but have a genetic substrate. It is a channelopathy with characteristic ECG changes (ST-segment elevation of >2 mm with a coved-type morphology in >1 right precordial lead) and an increased risk of sudden cardiac death (SCD), in the absence of gross structural heart disease [37,64]. Although the pathophysiology of BS is under investigation, it has been hypothesized that autonomic modulation may participate in the genesis of the syndrome. Abnormal norepinephrine recycling, identified in BS, is indicative of abnormal autonomic innervation causing ventricular tachyarrhythmias and sudden death at rest or during sleep and of typical ECG changes under pharmacological modulation of the myocardial autonomic tone [65]. 

In 1998, two case reports were published, describing the cases of two middle aged men with Brugada syndrome who underwent MIBG scintigraphy, to evaluate myocardial autonomic innervation. Nomura et al. observed a decreased accumulation or an unequal distribution of [^123^I]MIBG in part of the inferior wall, the apex and anterior wall of the left ventricle as well as an increased wash out in the inferior wall [66]. Similarly, Agostini et al. found defects of myocardial neuronal MIBG uptake on MIBG SPECT imaging in inferior, apical and septal walls [67]. Both researchers found no abnormality in myocardial blood flow and set the basis for the clinical utility of myocardial MIBG SPECT imaging in BS. In another case study from Japan no accumulation of MIBG was found anywhere throughout the heart in a 22-year-old patient with Brugada Syndrome who was resuscitated from cardiopulmonary arrest, confirming previous results [68].

Larger case control studies were conducted some years later. Wichter et al. investigated using of MIBG scibtigraphy, the uptake-1 in 17 patients with BS and in 10 controls, in the same age range, and quantitative 33-segment bull’s-eye analysis. They showed regionally reduced [^123^I]-MIBG uptake in 8 (47%) of 17 patients but in none of the control patients. Quantitative analysis showed segmentally decreased uptake in the inferior and septal left ventricular wall in patients with BS compared with control subjects. However, no correlation was found between MIBG uptake and the clinical characteristics of the study patients [69]. Besides clinical characteristics, another confounder that has been found to affect cardiac MIBG accumulation in BS is the shape of ST-segment elevation in ECG [70]. 

Postsynaptic and presynaptic myocardial sympathetic function in patients with BS has also been investigated by means of PET/CT. The cardiac autonomic nervous system of 9 patients with BS was examined non-invasively, using [^11^C]-HED to quantify myocardial pre- and postsynaptic sympathetic function and the nonselective β-blocker [^11^C]-CGP, by Kies et al. reported that norepinephrine recycling, as assessed by PET- [^11^C]-HED scintigraphy, was increased in patients with BS compared to the control- group, while postsynaptic β-adrenoceptor density, was found to be of the same levels in patients and control subjects, further supporting the hypothesis of an autonomic dysfunction in BS [71]. Future studies of the pre- and post-synaptic sympathetic and parasympathetic myocardial innervation are needed, to support these findings.

### 5.4. Long QT Syndrome

Long QT syndrome (LQTS) is a congenital myocardial repolarization disorder characterized by a prolongation of the QT interval on ECG and a propensity to ventricular tachyarrhythmias, which may lead to syncope, cardiac arrest, or sudden death. Certain criteria for the diagnosis have been proposed by the ESC in 2015 [37] The primary symptoms of LQTS, that can be either congenital or acquired, include palpitations, syncope, seizures, and sudden cardiac death. Based on genetic investigation, it was suggested that there is a plethora of common gene-polymorphisms associated with this condition that may confer susceptibility to the development of torsade de pointes in some individuals, particularly when specific drugs are being administered. It is also noticeable that the severe form of the disease is sporadic and its genetic correlation remains unclear. Furthermore, some polymorphisms have been shown to have regulatory properties that either enhance or counteract a particular mutation’s impact [72]. Increased sympathetic activation has been documented to be a key modifier for arrhythmogenesis in patients with long LQTS.

K Göhl et al. performed one of the original studies to investigate the hypothesis of a specific sympathetic imbalance, using MIBG. According to their results, uniform tracer uptake was observed to all healthy volunteers with somehow slight reduction of activity in the apex. All patients with QT greater than 440 msec (*n* = 5), all who had suffered from at least one episode of torsade de pointes, ventricular fibrillation or syncope (*n* = 5) and all symptomatic patients with QT prolongation (*n* = 4) had reduced or abolished MIBG uptakes in the inferior and inferior septal parts of the left ventricle, introducing the critical role of molecular imaging in identifying myocardial sympathetic dysinnervation in LQTS [73]. Further researchers attempted to correlate the findings of cardiac MIBG scans with the underlying LQTS genotype. Kies et al. performed MIBG SPECT in 28 LQTS patients. An abnormal MIBG scan was found in 17 of 28 LQTS patients (61%) with a tracer reduction mainly located in the anteroseptal segments of the left ventricle. This finding was independent of the genetic LQTS subtype [74].

Mazzadi et al. evaluated sympathetic nervous system using [^11^C]HED and PET in genotyped LQTS patients. [^15^O] H_2_O and [^11^C]HED PET studies were carried out in 11 patients (5 symptomatic) and 8 controls. Controls and patients showed similar sectorial perfusion, while most LQTS patients showed a localized and decreased pattern of [^11^C]HED retention. The larger number of heterogeneous sectors in symptomatic patients suggests that sympathetic function could play an amplifier role for severity of the disease [75].

## 6. Conclusions

Cardiac autonomic research remains an evolving area due to its crucial role in a group of life-threatening arrhythmias, while imaging can specify the severity of the disease and contribute as a therapeutic and prognostic tool. Radiotracer analogs of norepinephrine have been investigated extensively during the past 30 years and remain in widespread clinical use. [^123^I]-MIBG remains the most widely studied and used SPECT tracer, able to distinguish between normal hearts and hearts where the autonomic nervous system is disarranged, like in cardiomyopathies or in arrhythmogenic syndromes. Increased and homogenous cardiac uptake appears to have high negative predictive value for severe cardiac events, i.e., death and arrhythmias, and could be crucial in decision making. Future studies revealing robust associations between cardiac autonomous innervation disorders and ventricular arrhythmias are needed and could provide novel insights in selecting patients who are suitable candidates for device implantation, such as an ICD (implantable cardioverter defibrillator), CRT (cardiac resynchronization therapy), LVAD (left ventricular assist device), or cardiac transplantation. 

Cardiac autonomic using SPECT and PET is able today to extract vital information, visualizing and quantifying the underlying molecular aspects of cardiac disease, providing a perspective that other cardiac non-invasive methods cannot.
